# Association of Left Ventricular Diastolic Dysfunction With Cardiovascular Outcomes in Patients With Pre-dialysis Chronic Kidney Disease: Findings From KNOW-CKD Study

**DOI:** 10.3389/fcvm.2022.844312

**Published:** 2022-03-25

**Authors:** Sang Heon Suh, Tae Ryom Oh, Hong Sang Choi, Chang Seong Kim, Eun Hui Bae, Kook-Hwan Oh, Kyu Hun Choi, Yun Kyu Oh, Seong Kwon Ma, Soo Wan Kim

**Affiliations:** ^1^Department of Internal Medicine, Chonnam National University Medical School, Chonnam National University Hospital, Gwangju, South Korea; ^2^Department of Internal Medicine, Seoul National University Hospital, Seoul, South Korea; ^3^Department of Internal Medicine, College of Medicine, Institute of Kidney Disease Research, Yonsei University, Seoul, South Korea; ^4^Department of Internal Medicine, Seoul National University, Seoul, South Korea

**Keywords:** chronic kidney disease, heart failure, left ventricular diastolic dysfunction, cardiovascular outcome, all-cause mortality

## Abstract

**Background:**

The impact of left ventricular diastolic dysfunction (LVDD) on cardiovascular (CV) outcomes in patients with pre-dialysis chronic kidney disease (CKD) has been rarely unveiled. We here investigated the association of LVDD with CV outcomes and all-cause mortality in patients with pre-dialysis CKD.

**Methods:**

A total of 2,135 patients with pre-dialysis CKD from the Korean Cohort Study for Outcomes in Patients With Chronic Kidney Disease (KNOW-CKD) cohort were dichotomized by the absence or presence of LVDD, which was defined as the ratio of the early transmitral blood flow velocity to early diastolic velocity of the mitral annulus (*E*/*e*′) > 14.

**Results:**

Cox regression analysis revealed that LVDD was significantly associated with increased risk of composite CV events [adjusted hazard ratio (HR) 2.194, 95% confidence interval (CI) 1.486–3.240] and all-cause mortality (adjusted HR 1.830, 95% CI 1.168–2.869). Restricted cubic splines visualized stringent linear correlations of *E*/*e*′ with both composite CV events and all-cause mortality. In the sensitivity analysis only including the subjects with left ventricular ejection fraction ≥ 50%, LVDD was still significantly associated with adverse CV outcomes (adjusted HR 1.984, 95% CI 1.325–3.000) and all-cause mortality (adjusted HR 1.727, 95% CI 1.083–2.754), suggesting that the impact of LVDD on the outcomes in patients with CKD is independent of LV systolic function. Subgroup analyses revealed that the associations were not modified by various clinical contexts, such as age, sex, burden of comorbid conditions, body mass index, estimated glomerular filtration rate, and albuminuria.

**Conclusion:**

LVDD is independently associated with adverse CV outcomes and all-cause mortality in patients with pre-dialysis CKD.

## Introduction

Heart failure (HF) is a major cause of cardiovascular (CV) mortality in patients with chronic kidney disease (CKD) (1), which is divided into HF with preserved ejection fraction (HFpEF), HF with reduced ejection fraction (HFrEF), and HF with mid-range EF by left ventricular ejection fraction (LVEF) (2). No evident systolic dysfunction of the left ventricle is reported in ~30–50% of patients with symptomatic HF, in which cases left ventricular diastolic dysfunction (LVDD) plays a major role in the pathogenesis (3–5). The mechanism of LVDD so far is mainly attributed to left ventricular hypertrophy (LVH) with myocardial interstitial fibrosis, which in turn contributes to myocardial stiffness and impairment in diastolic relaxation (6).

The LVDD is prevalent in patients with CKD, where chronic hypertension (HTN) and anemia may promote the development of LVH (6). Previous studies reported that LVDD develops even in patients with early stages of CKD (7) and is present in more than 60% of patients with pre-dialysis CKD (8). It has been previously noted that renal insufficiency increases the risk of LVDD (9), aggravation of pre-existing HF, and all-cause mortality (10). Inversely, it has been also reported that LVDD is associated with adverse CV outcomes and all-cause mortality, especially among patients with end-stage renal disease (ESRD) (11–13). However, the impact of LVDD on long-term CV outcomes in patients with pre-dialysis CKD has been rarely unveiled.

As LVDD is prevalent even among patients with early-stage CKD (7, 8), we hypothesized that clinical consequences of LVDD may be present before the initiation of maintenance dialysis. Therefore, we here investigated the association of LVDD with CV outcome and all-cause mortality in patients with pre-dialysis CKD. To validate the impact of LVDD independent of left ventricular (LV) systolic function, we conducted sensitivity analysis including only those with preserved LVEF (LVEF ≥ 50%). In addition, to examine whether the association is modified by clinical contexts, we also performed a series of subgroup analyses.

## Methods

### Study Design

**Figure 1:**
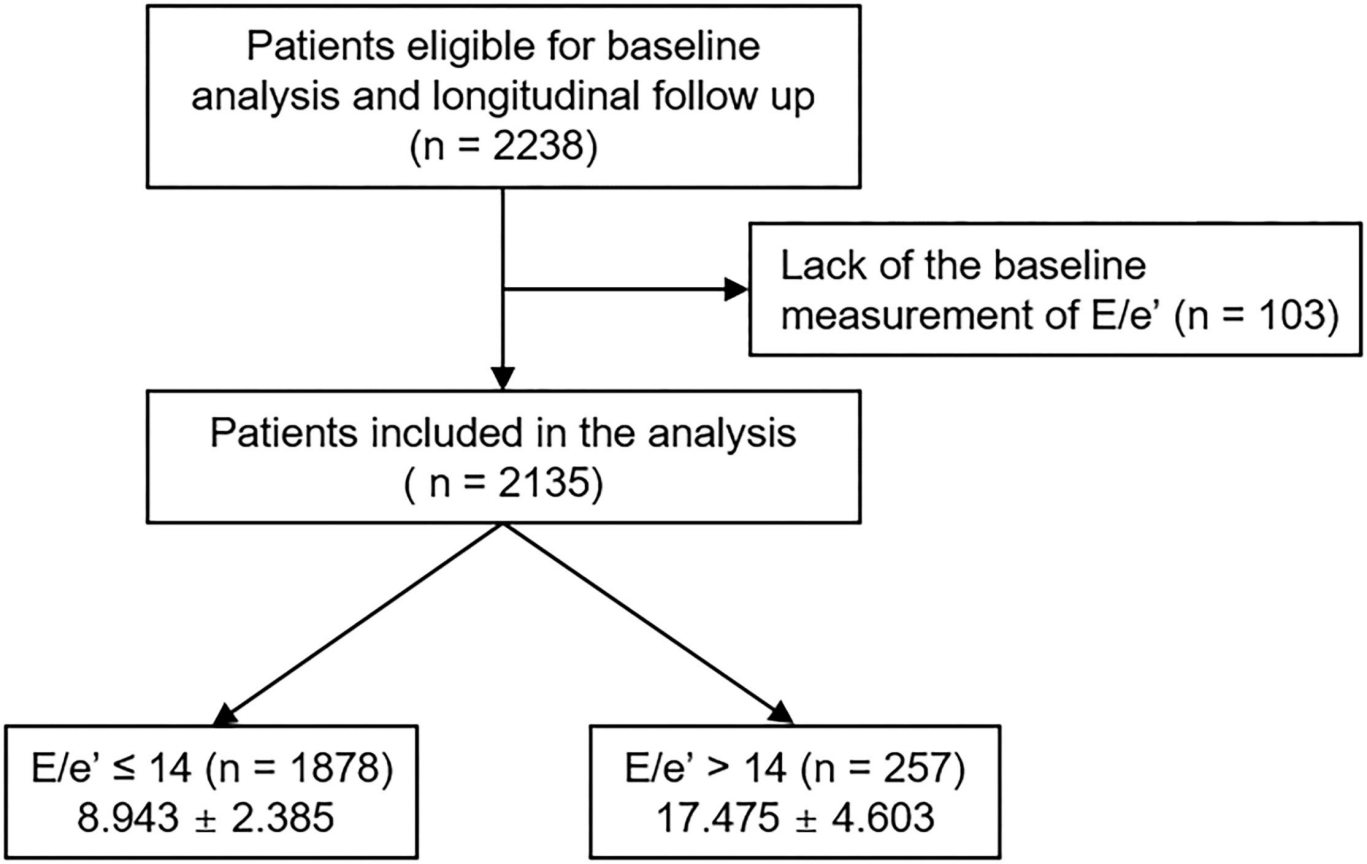
Flow diagram of the study participants. *E*/*e*′, ratio of the early transmitral blood flow velocity to early diastolic velocity of the mitral annulus.

The Korean Cohort Study for Outcomes in Patients With Chronic Kidney Disease (KNOW-CKD) is a nationwide prospective cohort study involving 9 tertiary-care general hospitals in Korea (NCT01630486 at http://www.clinicaltrials.gov) (14). Korean patients with CKD from stage 1 to pre-dialysis stage 5, who voluntarily provided informed consent were enrolled from February 2011 to January 2016. The study was conducted in accordance with the principles of the Declaration of Helsinki. The study protocol was approved by the institutional review boards of participating centers, including the Seoul National University Hospital, Yonsei University Severance Hospital, Kangbuk Samsung Medical Center, Seoul St. Mary's Hospital, Gil Hospital, Eulji General Hospital, Chonnam National University Hospital, and Busan Paik Hospital. All participants had been under close observation, and participants who experienced study outcomes were reported by each participating center. Among 2,238 participants who were longitudinally followed up, excluding those lacking the baseline measurement of the ratio of the early transmitral blood flow velocity to early diastolic velocity of the mitral annulus (*E*/*e*′), a total of 2,135 subjects were finally included for the analyses (Figure 1). The study observation period ended on March 31, 2020. The median follow-up duration was 5.987 years.

### Data Collection From Participants

Demographic information was collected from all eligible participants, including age, gender, comorbid conditions, smoking history, and medication history [angiotensin-converting enzyme inhibitor/angiotensin II receptor blockers (ACEi/ARBs), diuretics, number of anti-HTN drugs, and statins]. Trained staff members measured the height, weight, and waist circumference (WC) of study participants. Body mass index (BMI) was calculated as weight divided by the height squared. Systolic and diastolic blood pressures (SBPs and DBPs) were measured by an electronic sphygmomanometer after seated rest for 5 min. Venous samples were collected following overnight fasting, to determine hemoglobin, albumin, total cholesterol, low-density lipoprotein cholesterol (LDL-C), high-density lipoprotein cholesterol (HDL-C), triglyceride (TG), fasting glucose, high-sensitivity C-reactive protein (hs-CRP), 25-hydroxyvitamin D [25(OH) vitamin D], and creatinine levels at the baseline. The estimated glomerular filtration rate (eGFR) was calculated by the Chronic Kidney Disease Epidemiology Collaboration equation (15). CKD stages were determined by the Kidney Disease Improving Global Outcomes guidelines (16). Urine albumin-to-creatinine ratio (ACR) was measured in random, preferably second-voided, spot urine samples.

### Echocardiography

Complete two-dimensional M-mode and Doppler studies were performed *via* standard approaches by cardiologists at the participating hospitals who were blinded to the clinical data. M-mode examination was performed according to the American Society of Echocardiography guidelines (17). The recorded echocardiographic data were *E*/*e*′, LVEF, left atrial diameter, regional wall motion abnormality, valve calcification, LV posterior wall thickness, interventricular septum thickness, LV end-diastolic diameter, and LV end-systolic diameter. LV mass was determined using the Devereux formula (17). Left ventricular mass index (LVMI) was calculated by normalizing LV mass to body surface area (g/m^2^). LVDD was defined as *E*/*e*′ > 14 (9, 18, 19), although other indices, such as early mitral annulus *e*′ velocity (septal *e*′ < 7 cm/s or lateral *e*′ < 10 cm/s), left atrium volume index >34 ml/m^2^, and peak tricuspid regurgitation velocity >2.8 m/s, are also commonly used to evaluate LVDD (17).

### Study Outcomes

The outcomes of interest were composite CV events and all-cause mortality. Composite CV event included fatal and nonfatal coronary artery event [unstable angina (22 in 2,135, 1.0%), myocardial infarction (19 in 2,135, 0.9%), or coronary intervention/surgery (24 in 2,135, 1.1%)], hospitalization for HF, ischemic or hemorrhagic stroke, or symptomatic arrhythmia.

### Statistical Analysis

Continuous variables were expressed as mean ± standard deviation or median [interquartile range]. Categorical variables were expressed as the number of participants and percentage. The normality of distribution was ascertained by the Kolmogorov-Smirnov test. To compare the baseline characteristics according to *E*/*e*′ (*E*/*e*′ ≤ 14 vs. *E*/*e*′ > 14), the Student's *t*-test and χ^2^ test were used for continuous and categorical variates, respectively. In the primary analysis, the participants with any missing data were excluded for further analyses. To evaluate the association between LVDD and study outcomes, Cox proportional hazard regression models were analyzed. Patients lost to follow-up were censored at the date of the last visit. Models were constructed after adjusting for the following variables: Model 1 represents crude hazard ratios (HRs); Model 2 was adjusted for age, sex, Charlson comorbidity index, smoking history, medication (ACEi/ARBs, diuretics, number of antihypertensive drugs, and statins), BMI, SBP, and DBP; Model 3 was further adjusted for hemoglobin, albumin, fasting glucose, HDL-C, TG, 25(OH) vitamin D, hs-CRP, GFR, and spot urine ACR; and Model 4 was additionally adjusted for LVEF at the baseline. The results of Cox proportional hazard models were presented as HRs and 95% confidence intervals (CIs). Cumulative incidences of composite CV events and all-cause mortality were estimated using Kaplan-Meier analyses and were compared using the log-rank test. Restricted cubic splines were used to visualize the association between *E*/*e*′ as a continuous variable and HRs for study outcomes. To validate our findings, we performed sensitivity analyses. First, we excluded the subjects with eGFR <15 ml/min/1.73 m^2^, because those were relatively small in number and may exaggerate the association between LVDD and study outcomes due to far advanced CKD. Second, we excluded the subjects with eGFR ≥ 90 ml/min/1.73 m^2^, because those were considered close to normal kidney function and may not represent the CKD population well. Third, we excluded the subjects with LVEF < 50% to examine whether the association between LVDD and study outcomes is independent of LV systolic dysfunction. Finally, we replaced the missing values in primary analyses with multiple imputations and further conducted the Cox regression analyses. To examine whether the association of LVDD with the study outcomes is modified by certain clinical contexts, we conducted prespecified subgroup analyses. Subgroups were defined by age (<60 vs. ≥60 years), sex (male vs. female), Charlson comorbidity index (≤3 vs. ≥4), BMI (<23 vs. ≥23 kg/m^2^), eGFR (<45 vs. ≥45 ml/min/1.73 m^2^), and spot urine ACR (<300 vs. ≥300 mg/gCr). Two-sided *P*-values <0.05 were considered statistically significant. Statistical analysis was performed using SPSS for Windows version 22.0 (IBM Corp., Armonk, NY, USA) and R (www.r-project.org; R Foundation for Statistical Computing, Vienna).

## Results

### Baseline Characteristics

To describe the baseline characteristics, study participants were dichotomized by *E*/*e*′ (≤14 vs. >14) (Table 1). The mean follow-up duration was longer in the subjects with *E*/*e*′ ≤ 14. The mean age was higher in the subjects with *E*/*e*′ > 14. The frequency of male sex was higher in the subjects with *E*/*e*′ ≤ 14. Charlson comorbidity index, the frequency of diuretic use, medication of no <3 anti-HTN drugs, and statin medication, BMI, WC, SBP, but not DBP, were higher in the subjects with *E*/*e*′ > 14. Interestingly, the frequency of smoking history was higher in the subjects with *E*/*e*′ ≤ 14. Laboratory tests revealed that hemoglobin, albumin, HDL-C, 25(OH) vitamin D, and eGFR were lower in the subjects with *E*/*e*′ > 14, while TG, hs-CRP, and spot urine ACR were higher in the subjects with *E*/*e*′ > 14. Echocardiographic findings (Supplementary Table S1) revealed that, although LVEF was not significantly different between the two groups, left atrial diameter, LV posterior wall thickness, interventricular septum thickness, LV end-diastolic diameter, and LV end-systolic diameter were significantly higher in the subjects with *E*/*e*′ > 14. The frequency of regional wall motion abnormality and valve calcification was also significantly higher in the subjects with *E*/*e*′ > 14. Importantly, LVMI, which is a surrogate of LVH, was significantly higher in the subjects with *E*/*e*′ > 14. Therefore, with an exception of smoking history, these collectively indicate unfavorable underlying features in the subjects with *E*/*e*′ > 14.

**Table 1:** Baseline characteristics of study participants by *E*/*e*′.

	***E/e* ^′^**		***P*-value**
	**≤14**	**>14**	
Follow-up duration (year)	5.619 ± 2.165	5.128 ± 2.406	0.002
Age (year)	52.724 ± 12.216	60.051 ± 9.890	< 0.001
Male	1,164 (62.0)	141 (54.9)	0.028
**Charlson comorbidity index**			< 0.001
0–3	1,416 (75.4)	106 (41.2)	
4–5	436 (23.2)	141 (54.9)	
≥6	26 (1.4)	10 (3.9)	
Smoking history	893 (47.6)	100 (38.9)	0.009
**Medication**
ACEi/ARBs	1,607 (85.6)	221 (86.0)	0.856
Diuretics	555 (29.6)	117 (45.5)	< 0.001
Number of antihypertensive drugs ≥ 3	499 (26.6)	120 (46.7)	< 0.001
Statins	945 (50.4)	158 (61.5)	0.001
BMI (kg/m^2^)	24.407 ± 3.404	25.830 ± 3.205	< 0.001
WC (cm)	86.799 ± 9.649	91.564 ± 8.959	< 0.001
SBP (mmHg)	127.159 ± 15.697	133.718 ± 18.909	< 0.001
DBP (mmHg)	77.199 ± 10.962	75.906 ± 12.073	0.106
**Laboratory findings**
Hemoglobin (g/dL)	12.959 ± 2.000	11.956 ± 1.938	< 0.001
Albumin (g/dL)	4.195 ± 0.423	4.047 ± 0.444	< 0.001
Total cholesterol (mg/dL)	174.189 ± 39.338	172.574 ± 37.519	0.522
HDL-C (mg/dL)	49.759 ± 15.489	45.084 ± 13.937	< 0.001
LDL-C (mg/dL)	97.023 ± 32.116	93.706 ± 28.462	0.089
TG (mg/dL)	155.553 ± 96.21	170.853 ± 101.383	0.025
Fasting glucose (mg/dL)	109.335 ± 37.023	121.656 ± 52.717	< 0.001
25(OH) Vitamin D (ng/ml)	18.003 ± 7.919	16.232 ± 7.706	0.001
hsCRP (mg/dL)	0.600 [0.200, 1.600]	1.000 [0.400, 2.600]	0.044
Spot urine ACR (mg/gCr)	310.983 [62.444, 950.505]	734.152 [181.906, 2026.500]	< 0.001
eGFR (ml/min./1.73m^2^)	52.520 ± 30.581	35.803 ± 22.726	< 0.001
**CKD stages**			< 0.001
Stage 1	333 (17.0)	13 (5.1)	
Stage 2	380 (20.2)	24 (9.3)	
Stage 3a	316 (16.8)	34 (13.2)	
Stage 3b	394 (21.0)	59 (23.0)	
Stage 4	353 (18.8)	98 (38.1)	
Stage 5	102 (5.4)	23 (11.3)	

*Values for categorical variables are given as number (percentage); values for continuous variables, as mean ± standard deviation or median [interquartile range]. ACEi, angiotensin-converting enzyme inhibitor; ACR, albumin-to-creatinine ratio; ARB, angiotensin receptor blocker; BMI, body mass index; CKD, chronic kidney disease; Cr, creatinine; DBP, diastolic blood pressure; DM, diabetes mellitus; eGFR, estimated glomerular filtration rate; HDL-C, high-density lipoprotein cholesterol; hsCRP, high-sensitivity C-reactive protein; LDC-C, low-density lipoprotein cholesterol; SBP, systolic blood pressure; TG, triglyceride; WC, waist circumference*.

### Association of LVDD With Adverse CV Outcome and All-Cause Mortality in CKD

**Figure 2:**
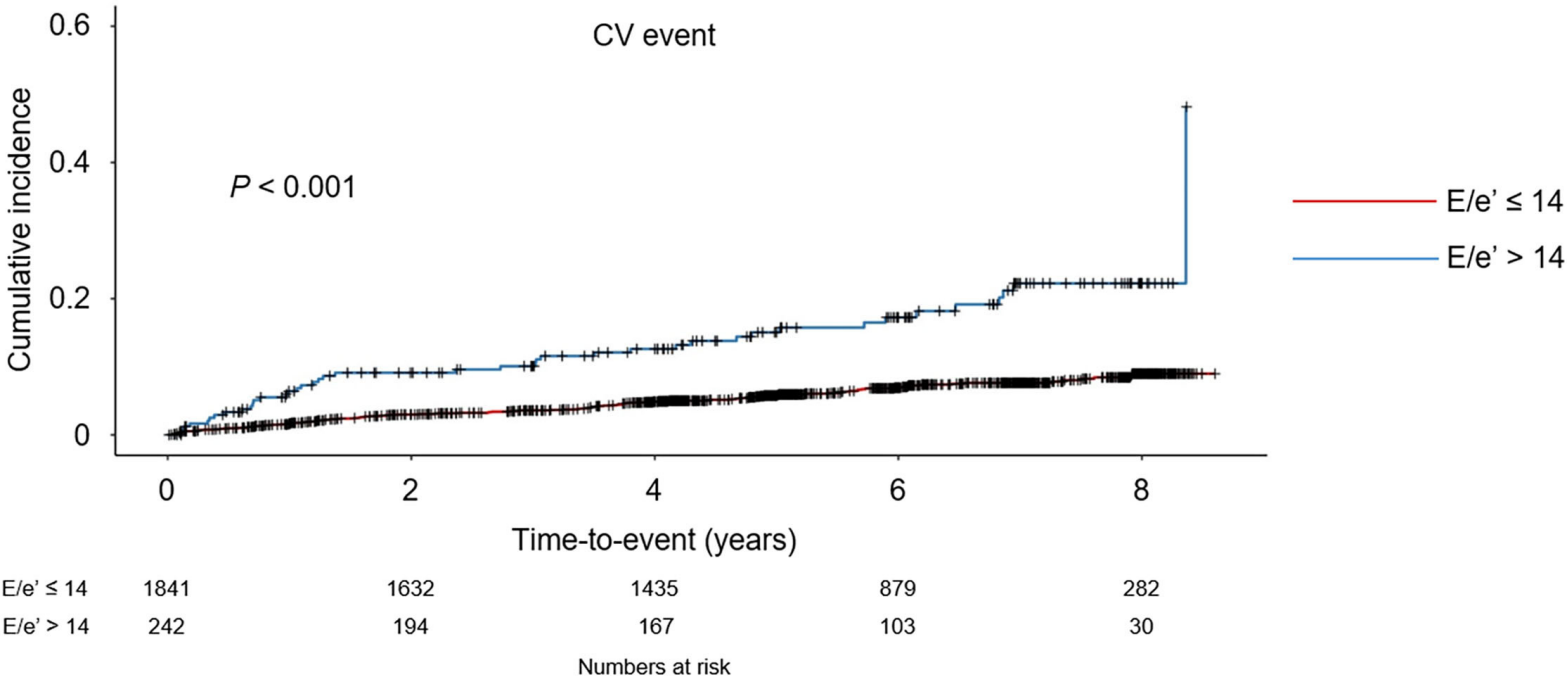
Kaplan-Meier analyses of cardiovascular (CV) event-free survival by *E*/*e*′. CV event-free survival curve is depicted. *P*-value determined using log-rank test. *E*/*e*′, ratio of the early transmitral blood flow velocity to early diastolic velocity of the mitral annulus.

**Figure 3:**
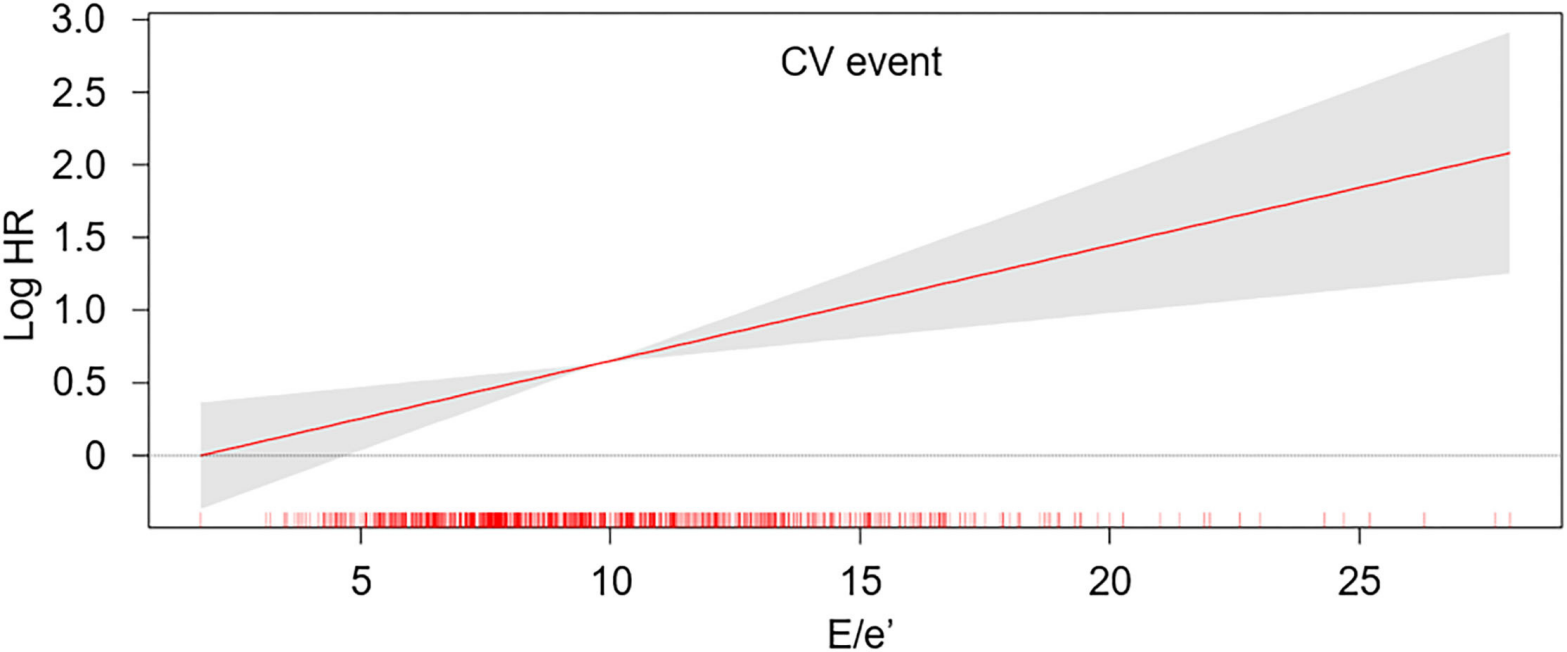
Restricted cubic spline of *E*/*e*′ on the risk of CV event. The adjusted HR of *E*/*e*′ as a continuous variable for the risk of composite CV event is depicted. The model was adjusted for age, sex, Charlson comorbidity index, smoking history, medication [angiotensin-converting enzyme inhibitor/angiotensin II receptor blockers (ACEi/ARBs), diuretics, number of antihypertensive drugs, statins], body mass index (BMI), systolic blood pressure (SBP), diastolic blood pressure (DBP), hemoglobin, albumin, fasting serum glucose, high-density lipoprotein cholesterol (HDL-C), triglyceride (TG), 25-hydroxyvitamin D (25(OH) vitamin D), high-sensitivity C-reactive protein (hs-CRP), glomerular filtration rate (GFR), spot urine albumin-to-creatinine ratio (ACR), ejection fraction (EF) at the baseline. *E*/*e*′, ratio of the early transmitral blood flow velocity to early diastolic velocity of the mitral annulus; HR, hazard ratio.

To compare the cumulative incidences of composite CV events and all-cause mortality, Kaplan-Meier analyses were conducted (Figure 2 and Supplementary Figure S1). The risk of composite CV events and all-cause mortality was significantly higher in the subjects with *E*/*e*′ > 14 (*P* < 0.001, determined using log-rank test). To determine the independent association of LVDD with study outcomes, Cox regression models were analyzed (Table 2). LVDD was significantly associated with the increased risk of composite CV events (adjusted HR 2.194, 95% CI 1.486–3.240) and all-cause mortality (adjusted HR 1.830, 95% CI 1.168–2.869). Restricted cubic splines visualized stringent linear correlations of *E*/*e*′ with both composite CV events and all-cause mortality (Figure 3 and Supplementary Figure S2).

**Table 2:** Cox regression analysis of left ventricular diastolic dysfunction (LVDD; *E*/*e*′ > 14) for clinical outcomes.

	**Events, *n* (%)**	**Model 1**		**Model 2**		**Model 3**		**Model 4**	
		**HR (95%CIs)**	***P-*value**	**HR (95%CIs)**	***P-*value**	**HR (95%CIs)**	***P-*value**	**HR (95%CIs)**	***P-*value**
Composite CV events	171 (8.0)	3.076 (2.127,4.449)	<0.001	2.250 (1.537, 3.219)	<0.001	2.156 (1.461, 3.182)	<0.001	2.194 (1.486, 3.240)	<0.001
All-cause mortality	132 (6.2)	2.846 (1.852, 4.371)	<0.001	2.207 (1.475, 3.301)	<0.001	1.867 (1.198, 2.910)	0.006	1.830 (1.168, 2.869)	0.008

*Model 1, unadjusted model; Model 2, model 1 + adjusted for age, sex, Charlson comorbidity index, smoking history, medication (ACEi/ARBs, diuretics, number of antihypertensive drugs, statins), BMI, SBP, and DBP; Model 3, model 2 + adjusted for hemoglobin, albumin, fasting serum glucose, HDL-C, TG, 25(OH) vitamin D, hs-CRP, GFR, and spot urine ACR; Model 4, model 3 + adjusted for EF at the baseline. CI, confidence interval; HR, hazard ratio*.

### Sensitivity Analysis

After excluding the subjects with eGFR < 15 ml/min/1.73 m^2^, who were relatively small in number and may exaggerate the association between LVDD and study outcomes due to far advanced CKD, the association of LVDD with adverse CV outcome (adjusted HR 1.838, 95% CI 1.194–2.829) and all-cause mortality (adjusted HR 1.693, 95% CI 1.036–2.766) was still valid (Supplementary Table S2). After excluding the subjects with eGFR ≥ 90 ml/min/1.73 m^2^, who were considered close to normal kidney function and may not represent the CKD population well, the association of LVDD with adverse CV outcome (adjusted HR 2.398, 95% CI 1.587–3.623) and all-cause mortality (adjusted HR 1.743, 95% CI 1.077–2.820) remained robust (Supplementary Table S3). In the analysis of the subjects with LVEF ≥ 50%, LVDD was still significantly associated with adverse CV outcome (adjusted HR 1.984, 95% CI 1.325**–**3.000) and all-cause mortality (adjusted HR 1.727, 95% CI 1.083–2.754) (Supplementary Table S4), suggesting that the impact of LVDD on the outcomes in CKD is independent of LV systolic function. Finally, after replacing the missing values with multiple imputations, LVDD was robustly associated with adverse CV outcome (adjusted HR 2.231, 95% CI 1.560–3.191) and all-cause mortality (adjusted HR 2.213, 95% CI 1.496–3.275) (Table 3).

**Table 3:** Cox regression analysis of LVDD (*E*/*e*′ > 14) for clinical outcomes using multiple imputations.

	**Model 1**		**Model 2**		**Model 3**		**Model 4**	
	**HR (95%CIs)**	***P-*value**	**HR (95%CIs)**	***P-*value**	**HR (95%CIs)**	***P-*value**	**HR (95%CIs)**	***P-*value**
Composite CV events	3.185 (2.278, 4.453)	<0.001	2.189 (1.541, 3.110)	<0.001	2.199 (1.541, 3.137)	<0.001	2.231 (1.560, 3.191)	<0.001
All-cause mortality	3.435 (2.362, 4.996)	<0.001	2.218 (1.503, 3.273)	<0.001	2.213 (1.496, 3.275)	<0.001	2.213 (1.496, 3.275)	<0.001

*Model 1, unadjusted model; Model 2, model 1 + adjusted for age, sex, Charlson comorbidity index, smoking history, medication (ACEi/ARBs, diuretics, number of antihypertensive drugs, statins), BMI, SBP, and DBP; Model 3, model 2 + adjusted for hemoglobin, albumin, fasting serum glucose, HDL-C, TG, 25(OH) vitamin D, hs-CRP, GFR, and spot urine ACR; Model 4, model 3 + adjusted for EF at the baseline. CI, confidence interval; HR, hazard ratio*.

### Subgroup Analysis

Subgroup analyses revealed that the association of LVDD with adverse CV outcome is not modified by age, sex, burden of comorbid conditions, BMI, eGFR, or albuminuria (Table 4). These clinical contexts did not alter the association of LVDD with all-cause mortality either (Supplementary Table S5).

**Table 4:** Cox regression analysis of LVDD (*E*/*e*′ > 14) for the risk of cardiovascular (CV) events in various subgroups.

	**Events, *n* (%)**	**Unadjusted**		**Adjusted**	
		**HR (95%CIs)**	***P* for interaction**	**HR (95%CIs)**	***P* for interaction**
Age < 60 years	71 (5.1)	3.829 (2.437,6.015)	0.511	2.988 (1.843,4.846)	0.308
Age ≥ 60 years	100 (13.4)	2.024 (1.044,3.925)		1.020 (0.484,2.151)	
Male	123 (9.4)	3.541 (2.287,5.483)	0.475	2.373 (1.487,3.787)	0.295
Female	48 (5.8)	2.314 (1.137,4.708)		1.856 (0.851,4.046)	
Charlson comorbidity index ≤ 3	82 (5.4)	3.015 (1.925,4.723)	0.624	2.253 (1.408,3.603)	0.341
Charlson comorbidity index ≥ 4	89 (14.5)	3.211 (1.662,6.203)		2.621 (1.262,5.443)	
BMI < 23 kg/m^2^	48 (7.1)	2.518 (0.329,19.269)	0.886	2.097 (0.209,21.081)	0.738
BMI ≥ 23 kg/m^2^	123 (8.5)	2.366 (1.619,3.458)		2.184 (1.465,3.256)	
eGFR ≥ 45 ml/min/1.73m^2^	66 (6.4)	3.581 (2.146,5.975)	0.984	3.051 (1.758,5.295)	0.588
eGFR < 45 ml/min/1.73m^2^	105 (9.5)	2.538 (1.473,4.373)		1.827 (1.007,3.313)	
Spot urine ACR < 300 mg/gCr	70 (7.3)	3.984 (2.368,6.703)	0.364	3.223 (1.822,5.700)	0.178
Spot urine ACR ≥ 300 mg/gCr	94 (8.6)	2.414 (1.417,4.111)		1.767 (0.991,3.150)	

*Models were adjusted for age, sex, Charlson comorbidity index, smoking history, medication (ACEi/ARBs, diuretics, number of antihypertensive drugs, statins), BMI, SBP, DBP, hemoglobin, albumin, fasting serum glucose, HDL-C, TG, 25(OH) vitamin D, hs-CRP, GFR, spot urine ACR, and EF at the baseline. ACR, albumin-to-creatinine ratio; CI, confidence interval; eGFR, estimated glomerular filtration rate; HR, hazard ratio*.

## Discussion

In this study, we demonstrated that LVDD is significantly associated with adverse CV outcomes and all-cause mortality in patients with pre-dialysis CKD. We also discovered that the association is robust even among the subjects with preserved LVEF, suggesting that the impact of LVDD on the CV outcomes in CKD is independent of LV systolic function.

It has been well documented that reduced eGFR increases the risks of all-cause mortality CV events, death, and hospitalization in patients with both HFpEF and HFrEF (20–23). Conversely, we here found that LVDD, a key feature of HFpEF, increases the risk of CV events and all-cause mortality in patients with pre-dialysis CKD. As the association of LVDD with adverse CV outcomes and all-cause mortality has been previously reported among the patients with ESRD (11–13), our finding is in line with the previous observations from the patients with ESRD. A cohort study of African Americans with hypertensive CKD reported a strong relationship between LVH and adverse cardiac outcomes (24), where the patients with eGFR of 20–65 ml/min/1.73 m^2^ were included for the analyses. As LVH contributes to the pathogenesis of LVDD, the findings indicate a possible association between LVDD and adverse CV outcomes in patients with CKD (24). More direct evidence to support the association between LVDD and adverse CV outcomes in patients with CKD was illustrated in another cohort study (25), where 89 out of 136 patients with CKD were at stage 5. The study demonstrated that the *E*/*e*′ ratio (*E*/*e*′ > 15) can predict mortality and CV events and all-cause mortality in patients with CKD (25). In addition to the previous reports, we here present compelling evidence for the association between LVDD and adverse CV outcomes in patients with CKD from a relatively large-scale analysis including the patients at all stages of CKD with various etiologies.

Despite the robust association of LVDD with CV events and all-cause mortality in patients with CKD, it is still unclear whether the direct cause of death in patients with LVDD is the exacerbation of HF. Although CV events account for up to 50% of deaths in patients with advanced CKD (26, 27), the cause of death is diverse, including coronary artery disease and stroke (28). In fact, two-thirds of LVDD cases result in ischemic heart disease in the general population (29), and the association of LVDD with coronary artery calcification has been previously reported (30–32). Therefore, it should be further addressed whether the direct cause of death in patients with LVDD and CKD is attributed to HF or whether LVDD accelerates preexisting atherosclerotic lesions.

## Limitations

There are several limitations in this study. First, we cannot determine the casual relationship between LVDD and study outcomes, due to the observational nature of this study. Second, as the formal definition of LVDD requires additional echocardiographic measures other than *E*/*e*′ (19), the definition of LVDD in this study (i.e., *E*/*e*′ > 14) is a simplified and modified one. Nevertheless, *E*/*e*′ is a feasible and reproducible index to assess the LV filling pressure (19, 33) and has been used to evaluate LVDD in the other cohort studies (9, 34, 35). Third, due to the operational definition of LVDD in this study, the grade of LVDD was not assessed. Accordingly, we were not able to address the impact of the severity of LVDD on the outcomes in the subjects. Interestingly, a meta-analysis reported a stepwise increase in all-cause mortality with CKD stages in patients with HF (36). It should be further addressed whether the severity of LVDD modifies the outcomes in patients with CKD. Fourth, echocardiography was examined in each center without central reading validation. Fifth, we are not able to determine the temporal sequence between LVDD and CKD in this study. Finally, as this cohort study enrolled only ethnic Koreans, a precaution is required to extrapolate the data in this study to other populations.

## Conclusion

We reported that LVDD is significantly associated with adverse CV outcomes and all-cause mortality in patients with pre-dialysis CKD. The association is robust even among the subjects with preserved LVEF, suggesting that the impact of LVDD on the CV outcomes in CKD is independent of LV systolic function.

## Data Availability Statement

The raw data supporting the conclusions of this article will be made available by the authors, without undue reservation.

## Ethics Statement

The study was conducted in accordance with the principles of the Declaration of Helsinki, and the study protocol was approved by the Institutional Review Boards of Participating Centers, including at Seoul National University Hospital, Yonsei University Severance Hospital, Kangbuk Samsung Medical Center, Seoul St. Mary's Hospital, Gil Hospital, Eulji General Hospital, Chonnam National University Hospital, and Pusan Paik Hospital. The patients/participants provided their written informed consent to participate in this study.

## KNOW-CKD

KNOW-CKD: Study Group Clinical Centers. Seoul National University, Curie Ahn, MD, Kook-Hwan Oh, MD, Dong Wan Chae, MD, Ho Jun Chin, MD, Hayne Cho Park, MD, Seungmi Lee, RN, Hyun Hwa Jang, RN and Hyun Jin Cho, RN. Yonsei University, Severance Hospital, Kyu Hun Choi, MD, Seung Hyeok Han, MD, Tae Hyun Yoo, MD, and Mi Hyun Yu, RN. Kangbuk Samsung Medical Center, Kyubeck Lee, MD, and Sooyeon Jin, RN. The Catholic University of Korea, Seoul St. Mary's Hospital, Yong-Soo Kim, MD and Sol Ji Kim, RN. Gachon University, Gil Hospital, Wookyung Chung, MD, Youkyoung Jang, RN, and Ji Hye Park, RN. Eulji University, Eulji General Hospital. Young-Hwan Hwang, MD, Su-Ah Sung, MD, and Jeong Ok So, RN. Chonnam University, Soo Wan Kim, MD and Ji Seon Lee. Inje University, Pusan Paik Hospital, Yeong Hoon Kim, MD, Sun Woo Kang, MD, and Yun Jin Kim, RN. Epidemiology and Biostatistics: Department of Preventive Medicine, Seoul National University College of Medicine, Byung-Joo Park, MD, Sue Kyung Park, MD, and Juyeon Lee. Coordinating Center: Medical Research Collaborating Center, Seoul National University Hospital, and Seoul National University College of Medicine, Joongyub Lee, MD, Dayeon Nam, RN, Soohee Kang, MSc, and Heejung Ahn, RN. Central Laboratory, Donghee Seo, MD, Lab Genomics, Korea, and Dae Yeon Cho, PhD, Lab Genomics, Korea. Biobank: Korea Biobank, Korea Centers for Disease Control and Prevention, Osong, Korea. Korea Center for Disease Control and Prevention, Dukhyoung Lee, MD, Hyekyung Park, MD (Project Officer), Eunkyeong Jung (Project Officer), Suyeon Jeong, Eunmi Ahn, and Sil-Hea Sung.

## Author Contributions

SS designed and helped in the data analysis and manuscript writing. SS, TO, and HC contributed to the conception of the study. SS and CK performed the data analyses and wrote the manuscript. EB, K-HO, KC, YO, and SK collected the data. SM and SK helped perform the analysis with constructive discussions. All authors contributed to the article and approved the submitted version.

## Funding

This study was supported by the Research Program funded by the Korea Centers for Disease Control and Prevention (2011E3300300, 2012E3301100, 2013E3301600, 2013E3301601, 2013E3301602, 2016E3300200, 2016E3300201, 2016E3300202, and 2019E320100), by the National Research Foundation of Korea (NRF) funded by the Korean Government (MSIT) (NRF-2019R1A2C2086276), and a Grant (BCRI21046) of Chonnam National University Hospital Biomedical Research Institute.

## Conflict of Interest

The authors declare that the research was conducted in the absence of any commercial or financial relationships that could be construed as a potential conflict of interest.

## Publisher's Note

All claims expressed in this article are solely those of the authors and do not necessarily represent those of their affiliated organizations, or those of the publisher, the editors and the reviewers. Any product that may be evaluated in this article, or claim that may be made by its manufacturer, is not guaranteed or endorsed by the publisher.
