# Supplementary material for: Association of Left Ventricular Diastolic Dysfunction With Cardiovascular Outcomes in Patients With Pre-dialysis Chronic Kidney Disease: Findings From KNOW-CKD Study
Source: Front Cardiovasc Med. 2022 Mar 25;9:844312. doi: 10.3389/fcvm.2022.844312 (PMC8990123; doi:10.3389/fcvm.2022.844312)
Supplement: Supplementary file 1 [file Data_Sheet_1.pdf]

- Supplemental Material -

Association of left ventricular diastolic dysfunction with cardiovascular outcomes in patients with pre-dialysis chronic kidney disease: findings from KNOW-CKD study

Sang Heon Suh, M.D., Ph.D.[1], Tae Ryom Oh, M.D., Ph.D.[1], Hong Sang Choi, M.D., Ph.D.[1], Chang Seong Kim, M.D., Ph.D.[1], Eun Hui Bae, M.D., Ph.D.[1], Kook-Hwan Oh, M.D., Ph.D.[2], Kyu Hun Choi, M.D., Ph.D.[3], Yun Kyu Oh, M.D., Ph.D.[4], Seong Kwon Ma*, M.D., Ph.D.[1], and Soo Wan Kim*, M.D., Ph.D.[1], on behalf of the Korean Cohort Study for Outcomes in Patients With Chronic Kidney Disease (KNOW-CKD) Investigators

[1]Department of Internal Medicine, Chonnam National University Medical School and Chonnam National University Hospital, Gwangju, Korea

[2]Department of Internal Medicine, Seoul National University Hospital, Seoul, Korea

[3]Department of Internal Medicine, College of Medicine, Institute of Kidney Disease Research, Yonsei University, Seoul, Korea

[4]Department of Internal Medicine, Seoul National University, Seoul, Korea

Corresponding authors

*Seong Kwon Ma, M.D., Ph.D., Department of Internal Medicine, Chonnam National University Medical School, 42 Jebongro, Gwangju 61469, Korea, Tel: +82-62-220-6579, Fax: +82-62-225-8578, Email: drmsk@hanmail.net

*Soo Wan Kim, M.D., Ph.D., Department of Internal Medicine, Chonnam National University Medical School, 42 Jebongro, Gwangju 61469, Korea, Tel: +82-62-225-6271, Fax: +82-62-220-8578, Email: skimw@chonnam.ac.kr

Table of Contents

Figure S1. Kaplan-Meier analyses of all-cause death-free survival by E/e’

Figure S2. Restricted cubic spline of E/e’ on the risk of all-cause mortality.

Table S1. Summary of echocardiographic findings of study participants by E/e’

Table S2. Cox regression analysis of LVDD (E/e’ > 14) for clinical outcomes excluding the subjects with CKD stage 5

Table S3. Cox regression analysis of LVDD (E/e’ > 14) for clinical outcomes excluding the subjects with CKD stage 1

Table S4. Cox regression analysis of LVDD (E/e’ > 14) for clinical outcomes in the subjects with LVEF ≥ 50%

Table S5. Cox regression analysis of LVDD (E/e’ > 14) for the risk of all-cause mortality in various subgroups

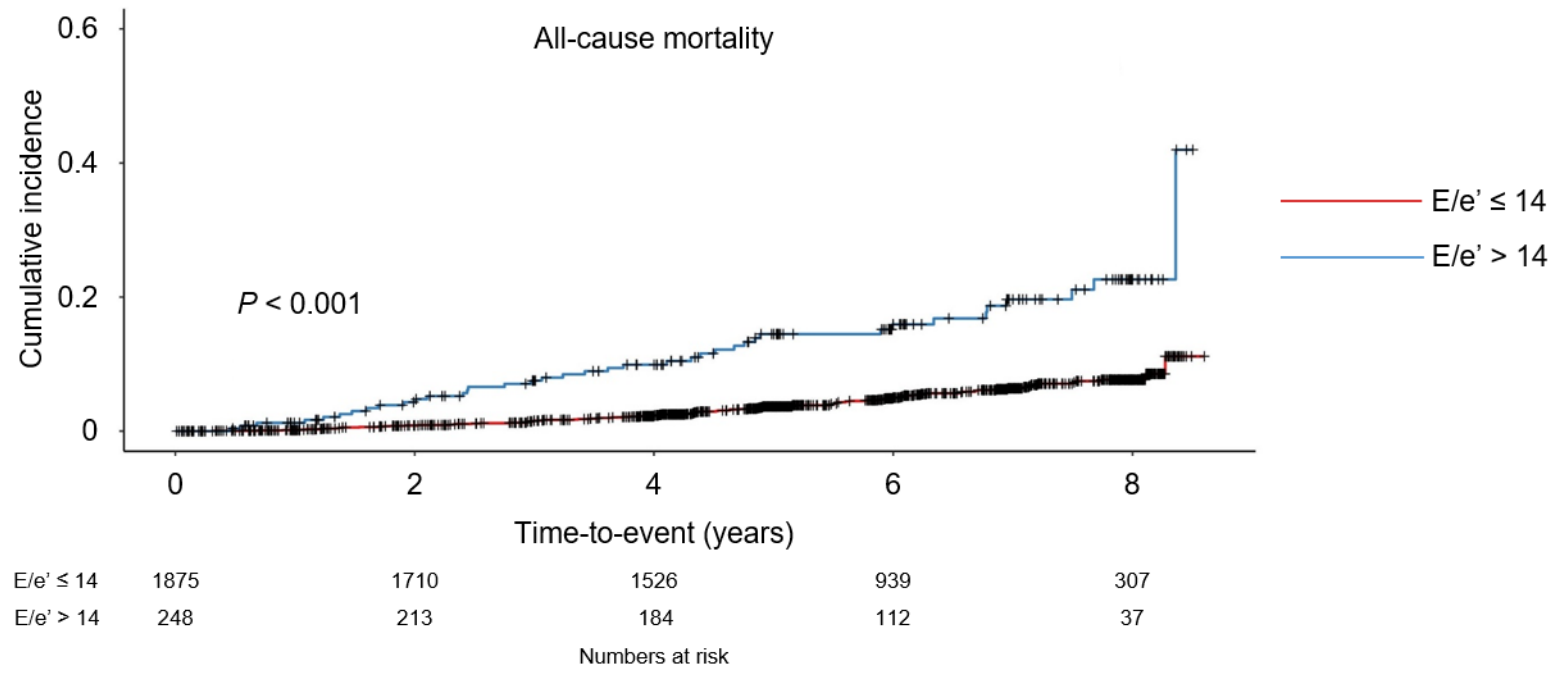

Figure S1. Kaplan-Meier analyses of all-cause death-free survival by E/e'

Note: All-cause death-free survival curve is depicted. *P* value by Log-rank test. Abbreviations: E/e', ratio of the early transmitral blood flow velocity to early diastolic velocity of the mitral annulus.

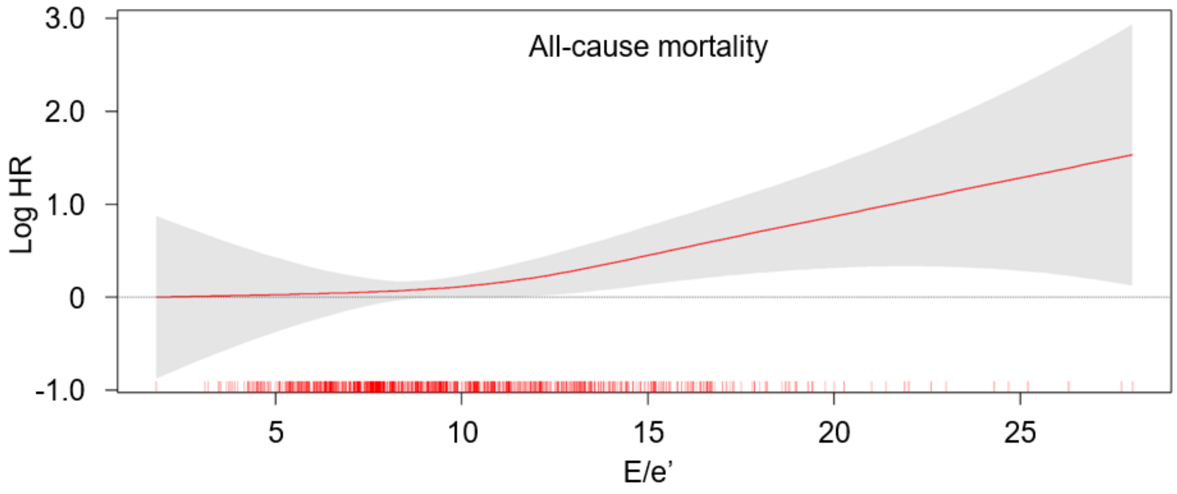

Figure S2. Restricted cubic spline of E/e’ on the risk of all-cause mortality.

Note: Adjusted HRs of E/e’ as a continuous variable for the risk of all-cause mortality is depicted. The model was adjusted for age, sex, Charlson comorbidity index, smoking history, medication (ACEi/ARBs, diuretics, number of antihypertensive drugs, statins), BMI, SBP, DBP, hemoglobin, albumin, fasting serum glucose, HDL-C, TG, 25(OH) vitamin D, hs-CRP, GFR, spot urine ACR, EF at the baseline. Abbreviations: E/e’, ratio of the early transmitral blood flow velocity to early diastolic velocity of the mitral annulus; HR, hazard ratio.

Table S1. Summary of echocardiographic findings of study participants by E/e'

	E/e'		
	≤ 14	> 14	*P* value
E/e'	8.943 ± 2.385	17.475 ± 4.603	< 0.001
LVEF (%)	64.071 ± 6.061	64.282 ± 7.572	0.670
LAD (mm)	37.231 ± 5.489	41.616 ± 5.980	< 0.001
RWMA	48 (2.6)	18 (7.1)	< 0.001
Valve Calcification	138 (7.4)	51 (20.2)	< 0.001
LVMI (g/m^2)	91.252 ± 23.274	111.826 ± 29.937	< 0.001
PWT (mm)	9.149 ± 1.517	10.187 ± 1.818	< 0.001
IVWT (mm)	9.235 ± 1.636	10.322 ± 2.036	< 0.001
LVEDD (mm)	48.513 ± 4.338	50.093 ± 5.297	< 0.001
LVESD (mm)	30.269 ± 4.065	31.412 ± 5.494	0.002

Note: Values for categorical variables are given as number (percentage); values for continuous variables, as mean ± standard deviation or median [interquartile range]. Abbreviations: E/e', ratio of the early transmitral blood flow velocity to early diastolic velocity of the mitral annulus; IVWT, interventricular wall thickness; LAD, left atrium diameter; LVEDD, left ventricular end-diastolic diameter; LVEF, left ventricular ejection fraction; LVESD, left ventricular end-systolic diameter;, LVMI, left ventricular mass index; PWT, posterior wall thickness; RMWA, regional wall motion abnormality.

Table S2. Cox regression analysis of LVDD (E/e' > 14) for clinical outcomes excluding the subjects with CKD stage 5

	Events, n (%)	Model 1		Model 2		Model 3		Model 4	
		HR (95%CIs)	*P* value	HR (95%CIs)	*P* value	HR (95%CIs)	*P* value	HR (95%CIs)	*P* value
Composite CV events	162 (8.1)	2.661 (1.775,3.99)	< 0.001	2.004 (1.363, 2.947)	< 0.001	1.792 (1.166,2.754)	0.008	1.838 (1.194,2.829)	0.006
All-cause mortality	113 (5.7)	2.699 (1.692, 4.308)	< 0.001	2.085 (1.350, 3.222)	< 0.001	1.737 (1.072, 2.816)	0.025	1.693 (1.036,2.766)	0.036

Note: Model 1, unadjusted model. Model 2, model 1 + adjusted for age, sex, Charlson comorbidity index, smoking history, medication (ACEi/ARBs, diuretics, number of antihypertensive drugs, statins), BMI, SBP, and DBP. Model 3, model 2 + adjusted for hemoglobin, albumin, fasting serum glucose, HDL-C, TG, 25(OH) vitamin D, hs-CRP, GFR and spot urine ACR. Model 4, model 3 + adjusted for EF at the baseline. Abbreviations: CI, confidence interval; HR, hazard ratio.

Table S3. Cox regression analysis of LVDD (E/e' > 14) for clinical outcomes excluding the subjects with CKD stage 1

	Events, n (%)	Model 1		Model 2		Model 3		Model 4	
		HR (95%CIs)	*P* value	HR (95%CIs)	*P* value	HR (95%CIs)	*P* value	HR (95%CIs)	*P* value
Composite CV events	164 (8.7)	3.440 (2.339, 5.060)	< 0.001	2.355 (1.604, 3.457)	< 0.001	2.386 (1.582, 3.600)	< 0.001	2.398 (1.587, 3.623)	< 0.001
All-cause mortality	129 (6.9)	2.676 (1.696,4.223)	< 0.001	2.073 (1.354,3.174)	< 0.001	1.807 (1.122,2.908)	0.015	1.743 (1.077,2.82)	0.024

Note: Model 1, unadjusted model. Model 2, model 1 + adjusted for age, sex, Charlson comorbidity index, smoking history, medication (ACEi/ARBs, diuretics, number of antihypertensive drugs, statins), BMI, SBP, and DBP. Model 3, model 2 + adjusted for hemoglobin, albumin, fasting serum glucose, HDL-C, TG, 25(OH) vitamin D, hs-CRP, GFR and spot urine ACR. Model 4, model 3 + adjusted for EF at the baseline. Abbreviations: CI, confidence interval; HR, hazard ratio.

Table S4. Cox regression analysis of LVDD (E/e' > 14) for clinical outcomes in the subjects with LVEF ≥ 50%

	Events, n (%)	Model 1		Model 2		Model 3		Model 4	
		HR (95%CIs)	*P* value	HR (95%CIs)	*P* value	HR (95%CIs)	*P* value	HR (95%CIs)	*P* value
Composite CV events	161 (7.7)	2.833 (1.927, 4.163)	< 0.001	2.102 (1.450, 3.048)	< 0.001	1.9857 (1.301, 2.944)	0.001	1.984 (1.325, 3.000)	< 0.001
All-cause mortality	124 (5.9)	2.750 (1.766, 4.282)	< 0.001	2.080 (1.370, 3.157)	< 0.001	1.703 (1.070, 2.713)	0.025	1.727 (1.083, 2.754)	0.021

Note: Model 1, unadjusted model. Model 2, model 1 + adjusted for age, sex, Charlson comorbidity index, smoking history, medication (ACEi/ARBs, diuretics, number of antihypertensive drugs, statins), BMI, SBP, and DBP. Model 3, model 2 + adjusted for hemoglobin, albumin, fasting serum glucose, HDL-C, TG, 25(OH) vitamin D, hs-CRP, GFR and spot urine ACR. Model 4, model 3 + adjusted for EF at the baseline. Abbreviations: CI, confidence interval; HR, hazard ratio.

Table S5. Cox regression analysis of LVDD (E/e’ > 14) for the risk of all-cause mortality in various subgroups

	Events, n (%)	Unadjusted		Adjusted	
		HR (95%CIs)	*P* for interaction	HR (95%CIs)	*P* for interaction
Age < 60 years	43 (3.1)	2.996 (1.779,5.045)	0.867	1.955 (1.125,3.396)	0.862
Age ≥ 60 years	89 (11.9)	2.636 (1.239,5.606)		1.44 (0.634,3.270)	
Male	98 (7.5)	2.321 (1.353,3.980)	0.291	1.467 (0.834,2.581)	0.156
Female	34 (4.1)	4.528 (2.138,9.589)		2.716 (1.195,6.171)	
Charlson comorbidity index ≤ 3	61 (4.0)	3.245 (1.959,5.375)	0.318	2.272 (1.338,3.859)	0.322
Charlson comorbidity index ≥ 4	71 (11.6)	2.212 (0.969,5.051)		1.344 (0.534,3.387)	
BMI < 23 kg/m^2	42 (6.3)	2.867 (0.370,22.222)	0.931	0.594 (0.041,8.609)	0.785
BMI ≥ 23 kg/m^2	89 (6.2)	2.212 (1.417,3.452)		1.838 (1.155,2.924)	
eGFR ≥ 45 mL/min/1.73m^2	29 (2.8)	3.890 (2.146,7.049)	0.277	2.734 (1.450,5.155)	0.171
eGFR < 45 mL/min/1.73m^2	103 (9.4)	2.048 (1.082,3.878)		1.458 (0.745,2.852)	
Spot urine ACR < 300 mg/gCr	50 (5.2)	3.953 (2.127,7.346)	0.529	2.915 (1.522,5.582)	0.134
Spot urine ACR ≥ 300 mg/gCr	79 (7.2)	2.221 (1.217,4.054)		1.520 (0.778,2.970)	

Note: Models were adjusted for age, sex, Charlson comorbidity index, smoking history, medication (ACEi/ARBs, diuretics, number of antihypertensive drugs, statins), BMI, SBP, DBP, hemoglobin, albumin, fasting serum glucose, HDL-C, TG, 25(OH) vitamin D, hs-CRP, GFR, spot urine ACR, EF at the baseline. Abbreviations: ACR, albumin-to-creatinine ratio; CI, confidence interval; eGFR, estimated glomerular filtration rate; HR, hazard ratio.
